# Investigation on the Curvature Correction Factor of Extension Spring

**DOI:** 10.3390/ma13184199

**Published:** 2020-09-21

**Authors:** P. S. Tan, Ali Akhavan Farid, Atefeh Karimzadeh, Seyed Saeid Rahimian Koloor, Michal Petrů

**Affiliations:** 1 Department of Mechanical, Materials and Manufacturing Engineering, University of Nottingham Malaysia Campus, Semenyih 43500, Malaysia; PengSam95@hotmail.com; 2 Institute for Nanomaterials, Advanced Technologies and Innovation, Technical University of Liberec, Studentska 2, 46117 Liberec, Czech Republic; michal.petru@tul.cz

**Keywords:** extension spring, finite element method, curvature correction factor, hook orientation, spring index, number of coils

## Abstract

The curvature correction factor is an important parameter in the stress calculation formulation of a helical extension spring, which describes the effect of spring wire curvature on the stress increase towards its inner radius. In this study, the parameters affecting the curvature correction factor were investigated through theoretical and numerical methods. Several finite element (FE) models of an extension spring were generated to obtain the distribution of the tensile stress in the spring. In this investigation, the hook orientation and the number of coils of the extension spring showed significant effects on the curvature correction factor. These parameters were not considered in the theoretical model for the calculation of the curvature correction factor, causing a deviation between the results of the FE model and the theoretical approach. A set of equations is proposed for the curvature correction factor, which relates both the spring index and the number of coils. These equations can be applied directly to the design of extension springs with a higher safety factor.

## 1. Introduction

Extension springs are extensively used in many industrial applications, such as in several parts and instruments in automotive, aerospace, robotics and machinery industries, in which spring failure could cause the failure of a whole system [1,2,3,4]. Extension springs are a part of the helical spring family. They are easily recognizable by the hooks on the ends of their body. Also known as tension springs, extension springs are used for tension applications to provide pulling force when extended. The hooks on the end of an extension spring’s body facilitate the transferring of tension loads to the body of the spring. Knowing the distribution of stress in the spring coil is necessary for the design of this part.

The stress in the coils of a loaded spring can be found through the calculation of shear stress due to torsion, which was described in a book by Wahl [5]. Before the introduction of Wahl’s equation, another method known as the approximate theory was used to calculate shear stress in the spring coil [5]. However, this method underestimates the stress on the inner side of the coil because of the rough assumptions about spring curvature [6]. Over the years, the effect of spring curvature on the shear stress augmentation has been studied through experimental methods [5,7,8,9], which led to proposing the curvature correction factor. The curvature factor acts as a design correction factor to increase the precision of the prediction of the stress required to cause spring failure under shear loading.

**Figure 1:**
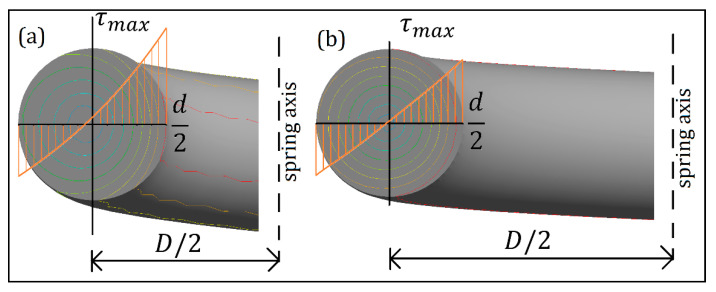
The effect of spring index toward distribution of stress: (**a**) small index, (**b**) large index.

Wahl’s equation depicted the curvature effect (Figure 1) as the increase in stress levels of the wire towards the inner radius [5]. The effect is described to be dependent on two parameters of the spring: the spring diameter (D) and the wire diameter (d). Springs with a larger D-to-d ratio will have a less prominent curvature effect as opposed to springs with smaller D-to-*d* ratio.

**Figure 2:**
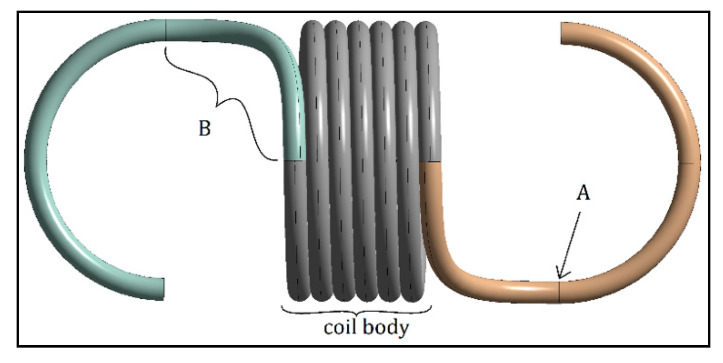
Extension spring critical points.

Wahl did not mention stress correction for the hook of an extension spring in his 1944 publication [5]. In a more recent publication, Budynas et al. [8] and the “Spring Research Association” [7] described another expression for the curvature correction factor known as the Bergsträsser factor ($K_{B}$). According to these researchers, extension springs have two critical points on the spring hooks, one due to the shear stress, located at section B, and one due to the bending stress, located at point A (Figure 2) [7,8]. Point B of the hook in the study of Budynas et al. was not shown clearly [8]. Therefore, point B is assumed to be the point of the arc section between the body and the hook at which maximum shear stress occurs.

In the case of extension springs, the orientation of the top and bottom hooks has generally not been discussed sufficiently in previous publications [10,11,12,13]. Illustrations often show that the two hooks are in opposing directions [5,14]. The position of the hooks is the relative offset angle between the two hooks. Some manufacturers offer extension springs where hooks are aligned, but usually they are in random positions. In theory, the hook’s orientation should not have any effect on the stress distribution. However, one of the hypotheses is that orientation of the hooks in the same plane will have an adverse effect on the stress distribution (lopsided stress distribution), resulting in higher stress values [15]. Previous publications [9,10,11,12,13,16,17,18] have not mentioned the correlation between hook orientation and the number of coils (N) against the stress correction factor.

Numerical methods, such as the finite element method, have been applied in previous research to design helical springs that eliminate the limitations of the theoretical and experimental methods in the calculation and measurement of the maximum stress in the different sections of the spring [19,20,21]. In these methods, the effects of various physical and mechanical parameters are studied using a valid simulation of the problem without spending much time on the testing of each parameter. In the review performed by Shimoseki et al. [22], it was also mentioned that the geometrical parameters of the springs have not been considered in the theoretical methods.

The difference between the resulting stress obtained through various equations proposed for the curvature correction factor and the lack of numerical works on the effect of geometrical parameters such as hook orientation on this factor are the main motivations of this study. Therefore, the purpose of this study is to investigate the effects of hook orientation and the number of coils on the stress distribution in the spring. For this purpose, the curvature correction factor was determined for various hook orientations and different numbers of coils through several finite element (FE) simulations of an extension spring under static structural conditions in ANSYS Workbench 17.2. The values obtained through the FE simulations were then compared to the theoretical methods published by mainstream texts.

## 2. Methods

The shear stress (*τ_max_*) in an extension spring was described by Wahl [5] as

(1) $$\tau_{max}=K\times \frac{8FD}{\pi d^{3}}$$

where K is the curvature correction factor, which accounts for the curvature effect of a helical spring.

*K* is described by Wahl as $K_{W}$ with the following equation [5]:

(2) $$K_{W}=\frac{4C-1}{4C-4}+\frac{0.615}{C}$$

where *C* is known as the spring index and obtained from *D/d*. The curvature correction factor proposed by Budynas et al. [8] and the “Spring Research Association” [7], which is known as the Bergsträsser factor, $K_{B}$, is expressed as

(3) $$K_{B}=\frac{4C+2}{4C-3}$$

Their publication also describes the stress calculation for point A and point B in Figure 2. Point A of the hook experiences combined loading of bending and axial loading, which is expressed by Budynas et al. [8] in the following form:

(4) $$\sigma_{a}=F\left[{\left(K\right)}_{a}\frac{16D}{\pi d^{3}}+\frac{4}{\pi d^{2}}\right]$$

whereby the curvature correction factor about point A, ${\left(K\right)}_{a}$ is expressed as

(5) $${\left(K\right)}_{a}=\frac{4C_{1}^{2}-C_{1}-1}{4C_{1}\left(C_{1}-1\right)},C_{1}=\frac{2r_{1}}{d}$$

Point B of the hook experiences torsional stress, where

(6) $$\tau_{b}={\left(K\right)}_{b}\frac{8FD}{\pi d^{3}}$$

whereby the curvature correction factor about point B, ${\left(K\right)}_{b}$, is expressed as

(7) $${\left(K\right)}_{b}=\frac{4C_{2}-1}{4C_{2}-4},\;C_{2}=\frac{2r_{2}}{d}$$

**Figure 3:**
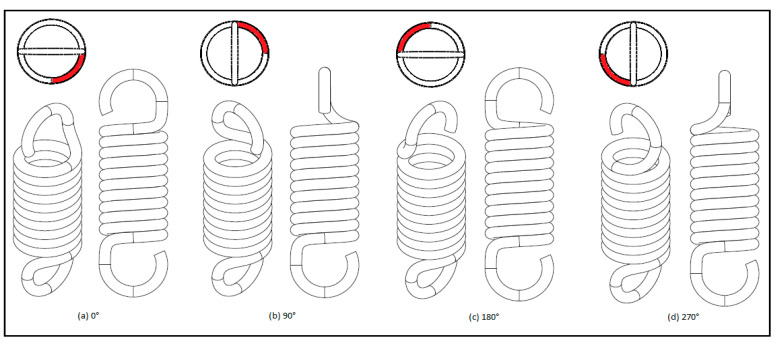
Hook orientations with varying degrees of offset (**a**) 0°, (**b**) 90°, (**c**) 180°, and (**d**) 270°; the bottom hook was taken as reference.

Hook orientation is a subset of multiple spring parameters including mean spring diameter (*D*), arc transition radii ($r_{1}$, $r_{2}$) and the length of the body coil [23]. The body coil length is a subset of two parameters: wire diameter (d) and number of coils (N) [23]. The definition of hook orientation in this report will be based on the difference in the angle of the hooks’ planes, as depicted in Figure 3, whereby offset is defined as degrees of rotation of the top hook relative to the bottom hook. If the planes of the top and bottom hooks are coplanar and the hooks face opposing directions, the spring is said to be at 0° offset. The spring model used for this study is a right-handed helix.

**Figure 4:**
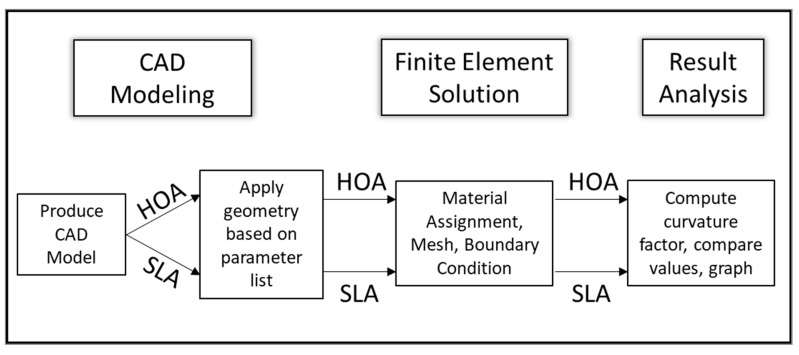
Methodology flow chart.

To investigate the effects of hook orientation and number of coils on the curvature correction factor, two sets of simulation, hook orientation analysis (HOA) and spring length analysis (SLA), were performed, as shown in Figure 4. The two analyses shared the same CAD model geometry and meshing methods and were applied with the same boundary conditions in the simulation model.

### 2.1. CAD Modeling

**Figure 5:**
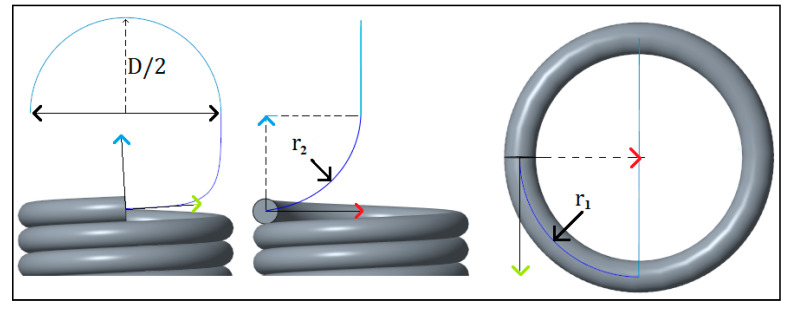
CAD modeling parameters for hook’s arc.

The modeling of the extension spring was based on illustrations by Budynas et al. [8] of a full twisted loop spring and were performed in PTC CREO 3.0. CAD (Las Vegas, NEV, USA) modeling. In this model, the spring body and spires were modeled as an integrated part, in which the body was created as a constant-pitch helical curve based on a given spring parameter, where the pitch was equivalent to the diameter of spring wire. A 3D curve with an upwards curvature radius of $r_{2}$ and a circular radius of $r_{1}$ were then sketched to form the arc (Figure 5), which leads to the hook of the spring, starting from where the helical curve ends. The line was then extended to resemble a hook, with a radius of D/2. Using the sweep function, the line was then extruded with a uniform circular cross-section of diameter *d* along all the sketched lines; thus, the CAD model was formed.

### 2.2. FE Simulation

The CAD model was simulated using commercially available ANSYS Workbench 17.2 software (Philadelphia, PA, USA), in which a static structural simulation model was used. The mechanical properties of stainless steel were assigned to the spring, of which the values of the shear modulus and Young’s modulus are shown in Table 1.

**Figure 6:**
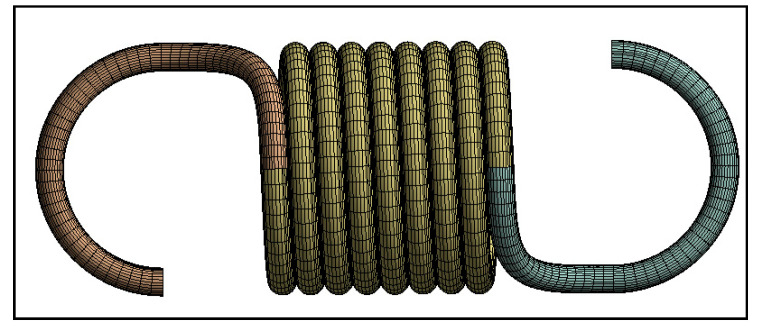
Meshed model.

The resultant meshed model (Figure 6) had about 175 k nodes and 40 k elements for a spring model with hook-to-hook length of 34.5 mm and with C = 8 and d = 1.5 mm. The numbers of nodes and elements changed according to spring parameters, such as N and D. The sweep method is perfect for a curved section with constant cross-section; thus, it was implemented onto the model.

**Figure 7:**
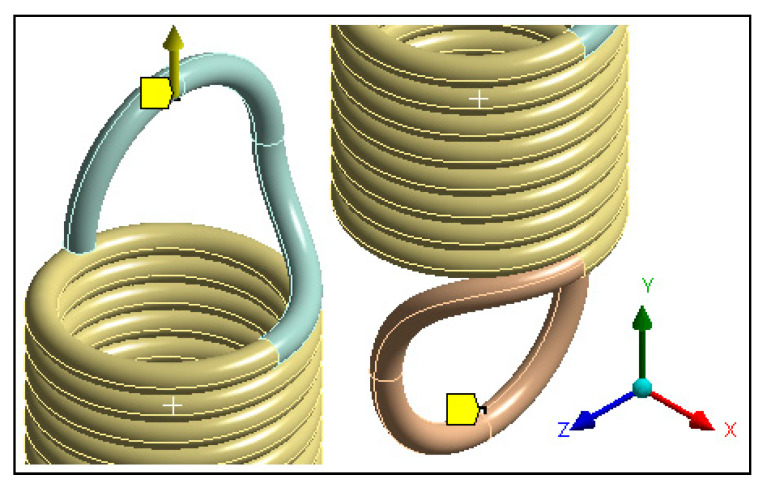
Boundary conditions.

A set of boundary conditions (BCs) was defined in the spring model (Figure 7), as listed in Table 2. The BCs were applied such that the bottom side of the spring was fixed to prevent any movement while the rotation parameters were free, while the loading was applied as displacement (not force). This research was a numerical study, considering displacement instead of force to apply deformation results in a smooth convergence, as the FE-based model initially calculated deformation of the structure and subsequently computed the force parameters. Such an assumption was considered in many basic studies in the literature [24,25,26].

Remote displacement was chosen instead of the regular displacement condition to allow the part to rotate freely about the Y axis (Figure 7). This was done to be able to mimic a real case of an extension spring under tension load, where the rotation about its axis is not constrained.

In the finite element method, the equilibrium equations should be solved to obtain the unknown displacements at each node. In the stress analysis, the general equation is

(8) [F]=[K][u]

where [*u*] is the displacement vector, [*K*] is the stiffness matrix and [*F*] is the load vector. The variational method was used to obtain the optimum values of constants by satisfying the internal compatibility and boundary conditions. In the elasticity problems, the total potential energy of the structure should be stationary with respect to the finite variations of displacement, expressed as follows:

(9) $$\frac{\partial W}{\partial a_{i}}=0$$

where *W* is the potential energy, and *a_i_* is *i*th degree of freedom including displacement or rotation.

(10) W=U+Ω

where *U* is the strain energy, and Ω is the load system’s potential. The strain energy is described as

(11) $$U=\;\frac{1}{2}\int {\left\{\varepsilon \right\}}^{T}\left[E\right]\left\{\varepsilon \right\}dV$$

where [*E*] is the matrix of the elastic constant, which describes the relation of stress and strain:

(12) {σ}=[E]{ε}

in which {σ} and {ε} are stress and strain tensors, respectively. The load system potential is defined by

(13) $$\Omega =-{\left\{u^{e}\right\}}^{T}\left\{F^{e}\right\}$$

By substituting Equations (11) and (13) into Equation (10), the total potential energy would be

(14) $$W=\;\frac{1}{2}\int {\left\{\varepsilon \right\}}^{T}\left[E\right]\left\{\varepsilon \right\}dV-{\left\{u^{e}\right\}}^{T}\left\{F^{e}\right\}$$

The vector of displacement in an element, {*u*}, is related by a shape function, [*N*], to the nodal displacement vector, $\left\{u^{e}\right\}$.

(15) $$\left\{u\right\}=\left[N\right]\left\{u^{e}\right\}$$

Therefore, the strain can be obtained from

(16) $$\left\{\varepsilon \right\}=\left\{\frac{du}{dx}\right\}=\left[\frac{dN}{dx}\right]\left\{u^{e}\right\}$$

The stress is then calculated by substituting the strain tensor into Equation (12). More information about the shape functions and the numerical methods for solving the above equations can be found in [27].

### 2.3. Simulation Analyses

One of the parameters that was varied through the simulations is spring index. For the purpose of simplification, D was kept constant, while d was varied, to produce the values of C between 4 and 12. In addition to that, the arc radii $r_{1}$ and $r_{2}$ were kept as the same value as D/2, which maintained a constant hook orientation angle.

#### 2.3.1. Hook Orientation Analysis (HOA)

Analysis of the effects of hook orientation against the curvature factor was done by changing the hook orientation from 0° to 360° in small increments. The modification of orientation was performed by increasing the number of coils in the body (*N*) from 10 to 11, in increments of 0.0625, which equates to increments in orientation of 22.5°, as shown in Table 3.

#### 2.3.2. Spring Length Analysis (SLA)

Analysis of the effects of the N against the curvature correction factor was done by changing N from 3 to 16 in increments of 1. Since *C* is one of the known variables for the curvature correction factor, the analysis of the spring length was performed in the preferred range of C from 4 to 12, indicated in Table 4.

## 3. Results and Discussion

### 3.1. Simulation Validation

The parameters obtained from the simulations are the maximum shear stress, bending stress and normal stress, directional deformation and reaction force. The maximum shear stress was measured for the body section and section B, while the bending and normal stresses were measured for the hook at section A. The measurement of these stresses was obtained by reading the maximum value from the data bar scale. The data bar scale range was limited to the area of interest only. For instance, the maximum stress value at the body section had its data bar coverage limited to the spring body. These maximum values were firstly noted down in a spreadsheet.

The FE correction factors for these three locations were obtained by substituting the maximum shear stress in the body and section B as well as the combination of bending and normal stresses into Equations (1), (4) and (6). The corresponding correction factors of the spring body and points A and B were then calculated by using Equations (3), (5) and (7), respectively. The FE simulation model was validated by comparing the computed correction factors obtained through the FE method with the values obtained through the theoretical method.

### 3.2. Results of Hook Orientation Analysis

**Figure 8:**
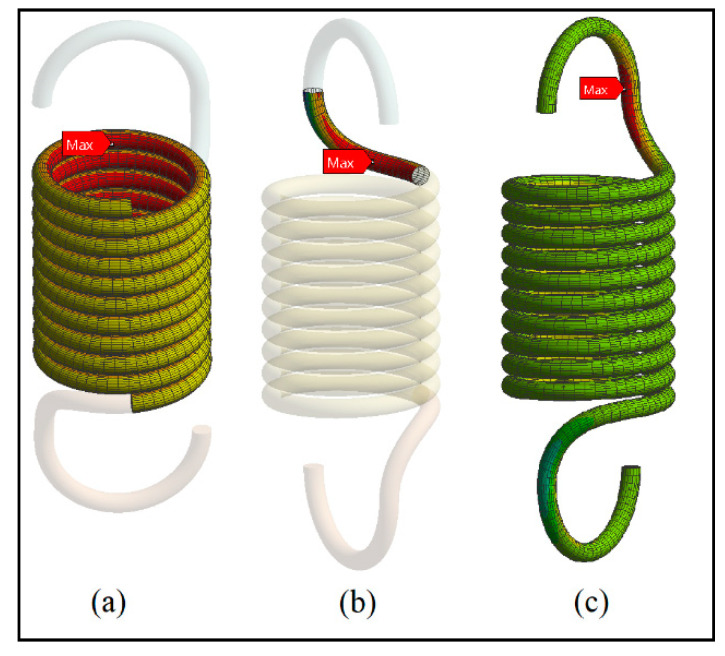
Locations of maximum stress in (**a**) the body, (**b**) point B and (**c**) point A.

The locations of maximum stress in the loaded extension spring body, point A and point B are shown in Figure 8. The locations of maximum stress from the FE simulation are validated by the research of Budynas et al. [8].

**Figure 9:**
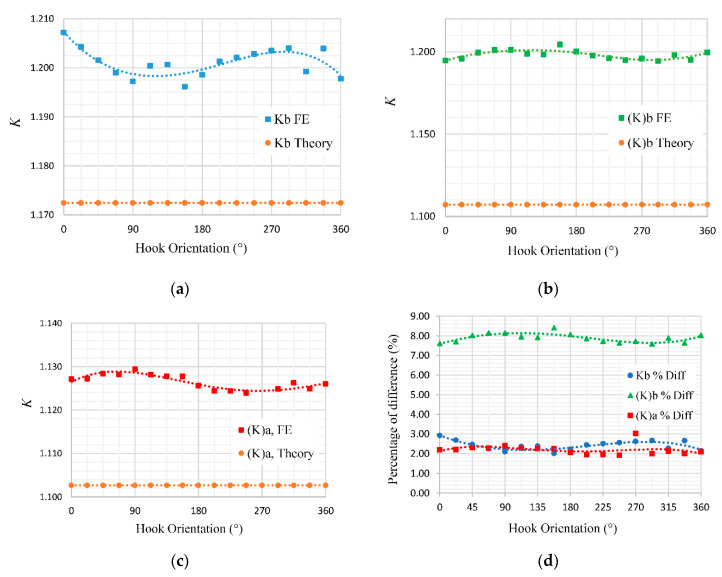
Effect of hook orientation on K_FE_ measured at (**a**) the spring body, (**b**) point B and (**c**) point A; (**d**) the percentage of difference between the correction factors obtained through the FE methods and the theoretical approach.

The curvature correction factor (*K*) was calculated at the locations shown in Figure 8 for different hook orientations, and the results are presented in Figure 9 in comparison with the results predicted by the theory. Based on the graphs plotted for HOA, the correction factor obtained from the FE simulation appears to have a sinusoidal pattern over a period of one revolution of a coil. The sinusoidal pattern carries a peak-to-peak value of about 0.005 for all three curvature correction factors calculated for the spring body, point A and point B.

A deviation of about 0.025 to 0.1 was observed in the correction factors of $K_{b,FE}$, ${\left(K\right)}_{b,FE}$ and ${\left(K\right)}_{a,FE}$, which was computed on the basis of the FE method as compared to the theoretical model. Higher values of correction factor indicate that the theoretical equation underestimated the stress levels in the extension springs. Such deviation leads us to suspect that there would be some other parameters affecting the values of the curvature correction factor. The initial assumption would be the length of the spring body, which identifies by the number of coils (*N*).

According to Figure 9d, the correction factor calculated from the FE approach for the arc section, i.e., ${\left(K\right)}_{b,FE}$, has a higher percentage of difference than other sections of the spring. The reason for the difference between the correction factors obtained from the FE simulation and the theory could be explained by the incompatibility of the theoretical equations with the type of hook end condition.

### 3.3. Results of Spring Length Analysis

**Figure 10:**
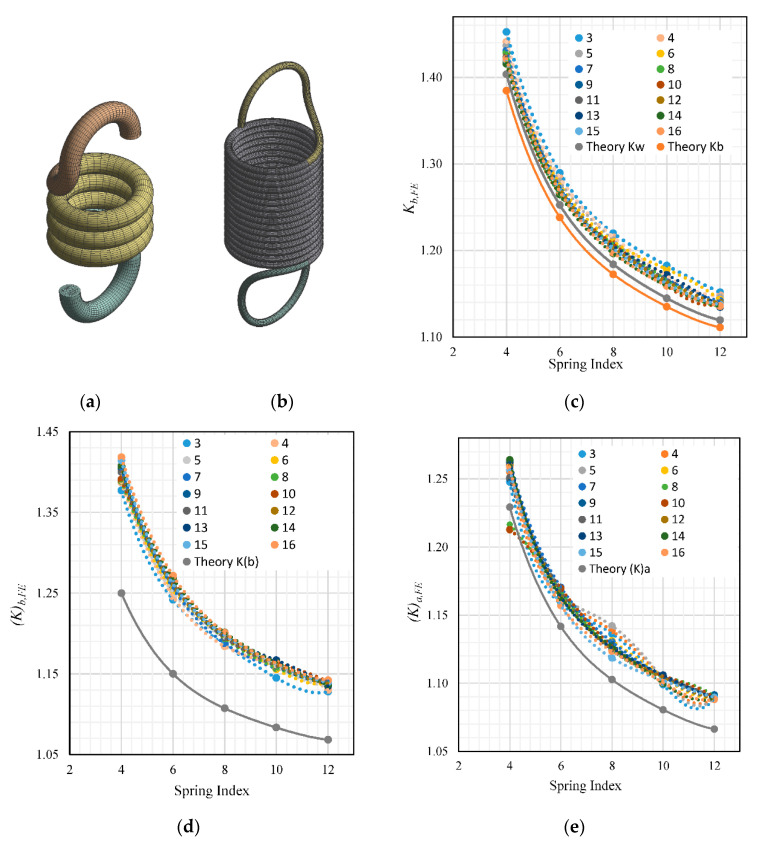
Change of parameter from (**a**) N = 3, C = 4 to (**b**) N = 16, C = 12; effects of spring index and number of coils against (**c**) $K_{b,FE}$, (**d**) ${\left(K\right)}_{b,FE}$, (**e**) ${\left(K\right)}_{a,FE}$.

The results of the SLA, which analyzed spring coils of N = 3~16 and C = 4~12 (Figure 10a,b), are shown in Figure 10c–e. The results agreed with the hook orientation analysis, whereby deviations were smaller for $K_{b,FE}$ and ${\left(K\right)}_{a,FE}$ for the lower spring indexes and larger for ${\left(K\right)}_{b,FE}$. As discussed in the previous section, the larger value of ${\left(K\right)}_{b,FE}$ deviation could be due to the incompatibility of the theoretical equation with the particular hook end condition that was modeled.

Interestingly, there are considerably more anomalies in Figure 10e, which threw off the overall trend of the linear shape of the curve. Such anomalies could be eliminated by reducing the element size of the mesh, but would come at the cost of longer processing time. The graphs plotted in Figure 10c–e show an observable trend, whereby with the change in N, the corresponding value of the curvature factor changes. The trend proves that there is indeed a correlation between N and the curvature factor. This brings the problem of calculation of the curvature correction factor to a functional equation with two independent variables, namely N and C, which could be represented as a surface plot, as opposed to the theoretical 2D curve where C is the only variable. The data can be presented well as a surface plot equation, whereby $K_{b,FE}$, ${\left(K\right)}_{b,FE}$ and ${\left(K\right)}_{a,FE}$ is a function of two independent variables, N and C, forming $K_{x}\left(N,C\right)$. Moreover, using Matlab’s curve-fitting tool, we could effectively formulate the function by importing the simulated data. The surface plots and their equations are described in the next section.

### 3.4. Surface Fitting

**Figure 11:**
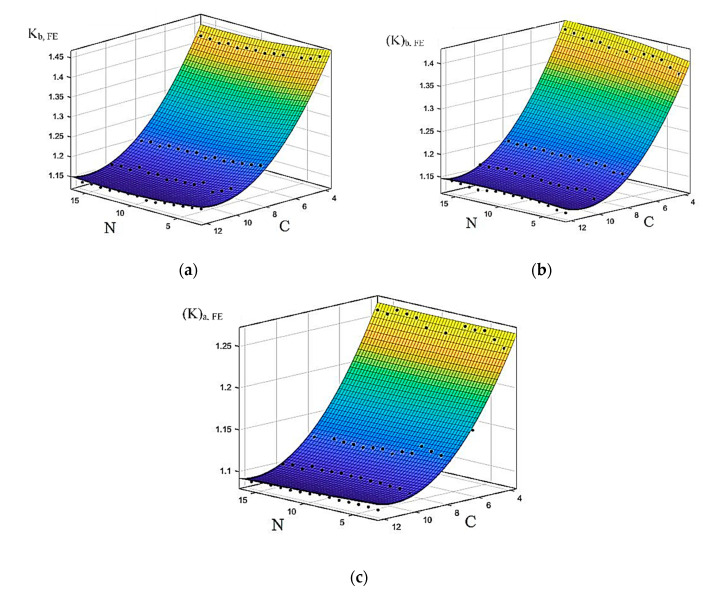
Surface plot based on SLA results for (**a**) K_(b,FE), (**b**) (K)_(b,FE) and (**c**) (K)_(a,FE).

The data collected from the SLA were drawn as surface plots, as shown in Figure 11, in which surface plot equations with a degree of 2 for both N and C were fitted. The corresponding equations are

(17) $$K_{b,FE}\left(N,C\right)=1.831-0.005755N-0.1157C+0.0001845N^{2}+0.000111NC+0.005043C^{2}$$

(18) $${\left(K\right)}_{b,FE}\left(N,C\right)=1.715+0.004355N-0.1059C-7.956\times 10^{-5}N^{2}-0.0001973NC+0.007481C^{2}$$

(19) $${\left(K\right)}_{a,FE}\left(N,C\right)=1.459-0.0001791N-0.06373C+1.609\times 10^{-5}N^{2}-4.568\times 10^{-5}NC+0.002807C^{2}$$

The R-squared values for the generated function equations (Equations (17)–(19)) have a minimum value of 0.9685, which could be improved by increasing the degree of polynomial for the two variables N and C but would introduce many terms into the equations.

The proposed surface plot equations were mapped on the basis of the data collected from the FE simulation as described in the methodology section. Therefore, the limits of these equations are bounded by the range of parameters used during the simulation, where *N* = 3~16 and *C* = 4~12.

Table 5 shows a set of extension springs with the same geometry and hook end condition undergoing static structural finite element analysis as described in the methodology section. The set of parameters in Table 5 has random values for N, C, D and d, which serves the purpose of validating the surface-fitted equation. The results show less than a 1% difference for most of the data that are within the limits of the functions. Hence, a higher degree of polynomial is not necessary. However, the equations may not be applicable for springs with the end condition different than the one described in the methodology (i.e., side loop, over-center loop, etc.). For example, the difference was above 5% for the spring with *N* = 6 and *C* = 15, which is out of the range of selection. Another set of analyses that involves other hook end conditions would have to be performed in order to formulate an appropriate set of equations for those particular conditions.

## 4. Conclusions

The effects of the hook orientation and the number of coils of a helical extension spring on the curvature correction factor were investigated by using the theoretical and finite element simulation methods. The results indicated fluctuation in the values of the curvature correction factor as the hook’s orientation changed from 0° to 360°. Further analysis of the influence of the number of coils indicated that there was a correlation between this parameter and the curvature correction factor, which was not mentioned in the previous research. A set of surface-fitted equations of two independent variables, the number of coils (*N*) and the spring index (C), was then proposed to be used in place of the theoretical equation for the calculation of the curvature correction factor for the three critical points of an extension spring. The proposed equations calculated the curvature correction factor of the extension springs more accurately than the classic equation, which can be applied to the precise design of the extension springs.

## Figures and Tables

**Table 1:** Material parameter—A227 hard drawn wire [8].

Shear Modulus, G (GPa)	80.0
Young’s Modulus, E (GPa)	197.9

**Table 2:** Boundary conditions applied for all simulations.

Remote Displacement	Top Hook	Bottom Hook
X-Component (mm)	0	0
Y-Component (mm)	1.5d	0
Z-Component (mm)	0	0
Rotation X	Free	Free
Rotation Y	Free	Free
Rotation Z	Free	Free

**Table 3:** Simulation parameters for HOA.

Spring Parameter	HOA
C	8
D (mm)	12
d (mm)	1.5
N	From 10 to 11
Number of Steps	17 (0°, 22.5°, …, 360°)
Orientation Increment (°)	22.5 per step

**Table 4:** Simulation parameter for SLA.

Spring Parameter	SLA
C	From 4 to 12
Number of Steps	2 (*C* = 4, 6, 8, …, 12)
D (mm)	12
d (mm)	1.5
N	From 3 to 16
Number of Steps	14 (*N* = 3, 4, 5, …, 16)
N Increment	1 per step

**Table 5:** Difference between curvature correction factor (*K*) obtained through FE method against surface-fitted equation.

Spring Parameter	Percentage of Difference (%)		
	$K_{b,FE}$	${\left(K\right)}_{b,FE}$	${\left(K\right)}_{a,FE}$
*N* = 8, *C* = 7.5 *D* = 18 mm, *d* = 2.4 mm	−0.04	−0.30	−0.12
*N* = 15, *C* = 7.5 *D* = 18 mm, *d* = 2.4 mm	−0.30	−0.35	−0.19
*N* = 6, *C* = 15 *D* = 18 mm, *d* = 1.2 mm	−7.97	−8.73	−5.08
*N* = 6, *C* = 11.5 *D* = 23 mm, *d* = 2.0 mm	0.20	0.12	−0.04
*N* = 9.5, *C* = 6.5 *D* = 9.75 mm, *d* = 1.5 mm	−1.20	−0.88	−0.52
*N* = 5.25, *C* = 10.75 *D* = 15.05 mm, *d* = 1.4 mm	0.25	1.11	0.36
